# Innate Immune Receptors and Defense Against Primary Pathogenic Fungi

**DOI:** 10.3390/vaccines8020303

**Published:** 2020-06-13

**Authors:** Theo N. Kirkland, Joshua Fierer

**Affiliations:** 1 Division of Infectious Diseases, Departments of Pathology and Medicine, School of Medicine, University of California San Diego, San Diego, CA 92037, USA; jfierer@ucsd.edu; 2 VA HealthCare San Diego, San Diego, CA 92161, USA

**Keywords:** innate immunity, receptors, fungi, pathogenic fungi, microbial immunity, CLR, TLR

## Abstract

The innate immune system is critical for natural resistance to all pathogenic microorganisms, including fungi. The innate response plays a vital role in resistance to infections before the antigen-specific immune response and also influences antigen-specific adaptive immunity. There are many different receptors for the innate immune response to fungi, and some receptors have been found to play a significant role in the response to human infections with opportunistic fungi. Most human infections are caused by opportunistic fungi, but a small number of organisms are capable of causing infections in normal hosts. The primary pathogenic fungi that cause invasive infections include *Blastomyces* spp., *Cryptococcus gattii*, *Coccidioides* spp., *Histoplasma* spp., and *Paracoccidioides* spp. In this review of innate immune receptors that play a role in infections caused by these organisms, we find that innate immunity differs between organisms.

## 1. Introduction

The innate immune response is one of the first lines of defense against infections. Innate immunity involves receptors for viruses, bacteria, fungi, and parasites and some of the receptors and mechanisms of resistance to microbial infections are shared, while others are unique to a class of organisms. In this review, we will focus on receptors important for innate immunity to fungi that can cause systemic infections in normal people.

### 1.1. Fungi

Fungi are eukaryotic organisms that are ubiquitous in the environment. By conservative estimates, there are more than one million different species of microfungi [1]. Human beings are exposed to many of these organisms on a daily basis, but rarely develop fungal infections because almost all fungi are adapted to live in soil or dead plant material and only a few can survive in animals. However, there are more than 3 million life-threatening invasive fungal infections per year, so fungal infections are a serious medical problem [2]. The fungi that cause human disease fall into three broad categories: those opportunistic fungi that cause disease in immunocompromised hosts, those that cause superficial disease on skin or mucosal surfaces (e.g., dermatophytes and *Candida* spp.), and those that cause invasive disease in apparently immunocompetent individuals. Most cases of deep-seated fungal infections are caused by opportunistic fungi, and the number of opportunistic fungal infections is growing steadily, partially because the number of immunocompromised patients is growing due to the increase in the use of immunosuppressive drugs and biologics and the HIV/AIDS epidemic. Opportunistic organisms are inhaled from the environment or, like *Candida* spp., are part of the normal intestinal flora.

In contrast, invasive fungal infections in previously healthy people are mostly caused by a small group of thermally dimorphic fungi that include *Histoplasma* spp., *Coccidioides* spp., *Blastomyces* spp., and *Paracoccidioides* spp. This group of filamentous dimorphic organisms are closely related members of the class Eurotiomycetes and order Onygenales [3] and these fungi are endemic in the Western Hemisphere. Up to half of the human population in certain highly endemic areas have been infected with one of these organisms, although only a smaller fraction had symptomatic disease, and most of the symptomatic infections are self-limited and do not require antifungal therapy. People are usually infected by inhaling infectious spores into their lungs, but the disease may first manifest itself in extrapulmonary sites because there can be hematogenous dissemination from the lungs. With the exception of *Cryptococcus neoformans* and *Cryptococcus gattii*, primary pathogenic organisms are dimorphic, growing as molds in the soil environment (at room temperature) and yeasts (or spherules) in the body at 37 °C. The intracellular forms are able to survive long enough in the host to elicit an immune response. A T-cell-mediated immune response is needed for resolution of disease, so people with quantitative or functional T-cell deficiencies frequently develop severe and sometimes fatal disease. Severe infections occur in apparently immunocompetent people who for unknown reasons do not mount an effective immune response. Since these organisms have adapted to cause infections in normal hosts, understanding their virulence factors and the host immune response to them is important. In this article, we will focus on the interaction of primary pathogenic fungi with the innate immune system, as this is both the first line of defense and it also informs the development of adaptive immunity.

### 1.2. Fungal Pathogen-Associated Cell Wall Products

The innate immune receptors primarily recognize fungi through the complex carbohydrates in their cell wall. Although all fungi have a thick carbohydrate cell wall containing chitin, β-(1,3)-glucans, β-(1,6)-glucans, and α-glucans, there are many differences in the composition and organization of the cell wall layers in different species [4]. In addition, the cell wall composition can change in some instances as the organism differentiates. Therefore, many different receptors for innate immunity to fungi are to be expected [5]. However, there are some commonalities in the fungal cell wall structure. Fungal cell walls consist of layers with different components. Most fungi have chitin as the deepest layer of the cell wall and a substantial amount of β-(1,3)-glucan above that layer. β-(1,3)-Glucan elicits a strong innate immune response and that layer is usually masked by β-(1,6)-glucan, mannan, glycoproteins (in *Candida* spp.), and α-(1,3)-glucan (in *Aspergillus fumigatus*, *Histoplasma* spp., and *Blastomyces dermatitidis*). *Aspergillus* conidia have hydrophobic rodlets and melanin in the outer layer, while the outer layer of the invasive hyphal form primarily consists of galactomannan. In addition, all of these organisms have glycoproteins of one kind or another in the outer layers that are often glycophosphatidylinositol (GPI)-linked and bound to the glucan layer in the cell wall. The cell walls of primary pathogenic fungi contain chitin and β-(1,3)-glucan, but the architecture and detailed composition of their wall layers have not been determined in many species. *Cryptococcus* spp. are a special case. They have a large capsule, primarily consisting of cross-linked, complex carbohydrate glucuronoxylomannan (GXM). The capsule can be much thicker than the cell wall [6] and is required for pathogenicity [7].

## 2. Innate Immunity

The innate immune system is an early part of the immune response to infection that triggers acute inflammatory responses and influences the development of acquired immunity. The innate immune system is critical for an effective host response because it acts as soon as infection occurs, without the delay needed for somatic gene rearrangements to occur that antigen-specific immune response requires. This type of immunity is triggered via pattern recognition receptors (PRRs), which are encoded by germ line genes that recognize pathogen-associated microbial products (PAMPs). There are more PRRs for fungi than for any other type of organisms [4]. Some of the cellular PRRs and their fungal PAMPs are listed in Table 1.

The receptors are expressed on many different cells of the innate immune system, and cytokines can modulate the expression of some receptors. To add to the complexity, a wide variety of responses are triggered by their engagement, depending on the complexity of the fungal surface, the size of the particle, and the density of β-glucan (clustering of Dectin-1 is needed to trigger activation) and the combination of receptors that are engaged. Some of the most prominent cells involved are macrophages and dendritic cells, but polymorphonuclear phagocytes, natural killer cells, invariant killer cells, and various kinds of epithelial cells also have receptors for fungal ligands and play roles in the innate immune response. Fungal PAMPs can stimulate PRRs to trigger a number of responses, including phagocytosis, respiratory burst, and the induction or inhibition of cytokine secretion.

There is a large amount of data from studies of some organisms, such as *Candida albicans*, but there is much less data about the role of the response to other fungi in vivo. Studies of human genetic mutations of the innate receptors for fungi and their role in fungal diseases are the most relevant to understanding fungal infections in human beings, but a much smaller number of human studies have been done.

## 3. Innate Immune Receptors

Many fungi interact with both C-type lectin receptors (CLRs) and toll-like receptors (TLRs). The fungal PAMPs recognized by C-type lectin receptors and TLRs are shown in Table 1. The exact mechanism of those interactions is unknown, but activation of both pathways usually leads to increased production of protective cytokines. Not all these interactions take place at the plasma membrane. For instance, Dectin-1 phagosomes also include TLR-9, which could enable endosomal recognition of fungal CpG DNA after phagocytosis and killing of the ingested fungus [19].

### 3.1. C-Type Lectin Receptors

**Figure 1:**
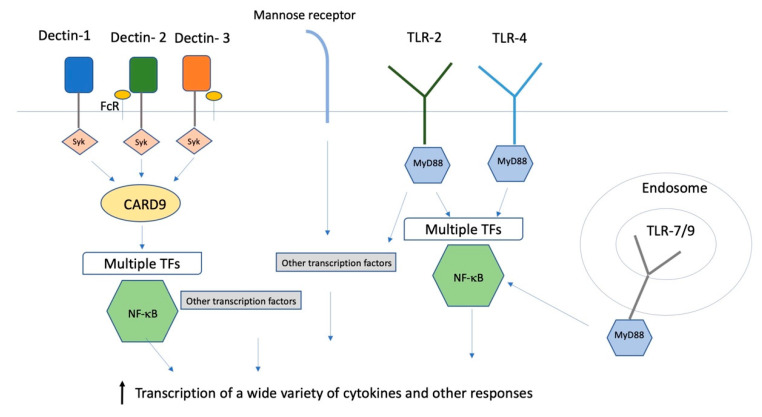
Innate immune receptors and transcription factors. A simplified schematic summary of some of the receptors and intracellular transcription pathways for the innate immune response to fungi. FcR: Fc receptor; TLR: toll like receptor; syk: signal transduction kinase; MyD88: Myeloid differentiation primary response 88; CARD9: caspase recruitment domain family, member 9; TFs: transcription factors.

C-type lectin receptors (CLRs) are membrane-localized signaling receptors expressed primarily on myeloid cells. They recognize polysaccharides that are not self, but are found on fungi and some bacteria. All of the CLRs that recognize fungi (Dectin-1, Dectin-2, mannose receptor (MR), dendritic cell-specific intercellular adhesion molecule-3-grabbing non-integrin (DC-SIGN), and macrophage inducible Ca^2+^-dependent lectin receptor toll-like receptors (Mincle)) are capable of inducing or modulating T_H_1 and T_H_17 responses, both of which are considered to be crucial for antifungal adaptive immunity [20]. Signaling through those receptors results in the binding of signal transduction kinase (Syk) to intracellular tyrosine motifs expressed either on the lectin receptors themselves or on the common Fc- γ receptor (FcRγ) that is the co-receptor for several CLRs, and that in turn activates transcription factors mitogen-activated protein kinase (MAPK) and nuclear factor of activated T cells (NFAT), and through the protein kinase Cδ−caspase recruitment domain family, member 9 -B cell lymphoma 10-mucosa-associated lymphoid tissue (PKCδ-CARD9-Bcl10-MALT1) axis, the transcription factor nuclear factor kappa light chain enhancer of activated B cells (NF-κB) Figure 1). Transcriptional activation has many outcomes, including phagocytosis, cytokine/chemokine secretion, and production of inflammatory lipids.

Dectin-1 (Clec7a) is a glycoprotein that is the primary recognition protein for β-(1,3) glucan. It is a small, type II membrane receptor with an extracellular C-type lectin-like domain fold and a cytoplasmic domain with a partial immunoreceptor tyrosine-based activation motif [21]. It is the only non-calcium dependent C-type receptor. Expression can be both positively and negatively regulated by cytokines. The gene is alternatively spliced in human and mouse cells, producing full-length and truncated proteins that are missing part of the stalk, as well as forms that are not exported and are of uncertain significance. Soluble β-glucans are inhibitors of signaling, and the larger β-(1,3)-glucan particles stimulate responses more effectively, presumably because they cluster the receptors, which results in exclusion of the regulatory tyrosine phosphatases CD45 and CD148 [22].

Dectin-1 signaling is very important for resistance to mucosal *Candida* infections in human beings. A stop mutation in Dectin-1 eliminates the expression of the lectin-binding domain and homozygotes have a mild form of mucocutaneous candidiasis, confirming the importance of Dectin-1 in resistance to that fungus [23]. Homozygous mutation of CARD9 (caspase recruitment domain family, member 9), which is involved in signaling through all the CLRs, leads to a severe form of mucocutaneous candidiasis and dermatophyte infections in humans, as well as invasive infections due to *Candida* and other fungi [24,25].

### 3.2. Toll-Like Receptors

Toll-like receptors (TLRs) are a family of at least 10 type I transmembrane PRR proteins with extracellular leucine-rich repeats that bind a variety of PAMPs and activate protective host responses to pathogens [26,27]. The receptors recognize many structural components of pathogens, including lipopolysaccharides, lipopeptides, polysaccharides, RNA, and DNA. Some TLRs are in the plasma membrane and others are found in the endosomes. TLRs can form heterodimers with each other or other types of PRRs. Once ligand binding has occurred, Myeloid differentiation primary response 88(MyD88) is a central adaptor protein that triggers a cascade of phosphorylation events that results in the activation of NF-κB and other transcription factors, resulting in increased transcription of a number of cytokines (Figure 1). MyD88 is also involved in signal transduction for the cytokine receptors for IL-1 and IL-18, so a response that requires MyD88 need not involve TLRs. Furthermore, TLRs can interact with CLRs to increase the inflammatory responses to fungi.

## 4. Innate Receptors for Pathogenic Fungi

We will discuss four of the most common primary pathogenic organisms that have been studied in some depth.

### 4.1. Blastomyces spp.

*Blastomyces* spp. grow as mycelia in the soil. When spores are inhaled by human beings and other animals, they differentiate into yeasts [28]. *B. dermatitidis* and other closely related species of *Blastomyces* are endemic along the major waterways of the northeastern and midwestern parts of the U.S. and neighboring Canadian provinces. In recent years, there have been reports of scattered infections in other parts of the world including sub-Saharan Africa and South Asia, but it is likely that they are due to other genera [29]. The skin lesions of blastomycosis were the first manifestation of this infection to be recognized, but we now know that most people are infected by inhalation. There have been a few epidemiological studies to establish the prevalence of blastomycosis in the largely rural communities that are at risk. Most infections are self-limited [30].

#### 4.1.1. BAD1 Receptors

BAD1 is a protein that is expressed by the yeast phase of the organism and is required for pathogenicity [31]. The protein is both secreted by and bound to the yeast cell surface. It promotes adhesion of the yeast to macrophages and inhibits TNF-α production by macrophages [31,32]. Both CD14 and complement receptor 3 (CR3) are receptors for BAD1 [33]. Although both receptors inhibit phagocytosis of the yeast and soluble BAD1-induced TNF-α suppression in vitro, the two receptors had very different effects on resistance to infection in vivo. CR3^−/−^ mice were highly resistant to infection, while CD14^−/−^ mice were no different than controls.

#### 4.1.2. CLR

Klein and his colleagues have reported that acquired immunity to *B. dermatitidis* required T_H_17 cells [34]. They have also studied the innate immune receptors and downstream molecules that are required for the development of a T_H_17 response [35]. They found that CARD9^−/−^ mice could not control infection (even when challenged with a BAD1 deletion hypovirulent mutant), and the mutant mice did not respond to immunization with killed *B. dermatitidis* yeast, indicating that a CLR was required for both innate and acquired immunity. CARD9 was also required for the differentiation of T_H_17 cells, but not expansion of antigen-specific T cells. The CLR receptor that they identified was Dectin-2. In *B. dermatitidis*, Dectin-2 recognizes a glycoprotein, designated Bl-Eng2, that is present on the yeast cell surface, rather than a complex carbohydrate [36]. This glycoprotein ligand is a more potent ligand than other ligands previously described in *Malassezia* spp. or *Mycobacterium tuberculosis*. Bl-Eng2 stimulates mouse bone marrow-derived dendritic cells (BMDCs) and human polymorphonuclear cells (PMNs) to produce IL-6, a critical cytokine for resistance to *B. dermatitidis*. FcRγ/Syk and CARD9 are also required for the development of a protective T_H_17 response to *B. dermatitidis* in mice, but Mincle is not.

The innate receptor Dectin-3 is also required for the development of protective immunity [37]. Mice lacking the Dectin-3 receptor did not make as vigorous a T_H_17 response to immunization as control animals. However, a direct comparison of the roles of Dectin-2 and Dectin-3 in acquired immunity showed that loss of Dectin-2 has a more profound impact on T_H_17 cell differentiation than that of Dectin-3.

The cell wall of *B. dermatitidis* contains exposed mannan, so the role of the mannose receptor was investigated and it was found to play a role in the T_H_17 response to *B. dermatitidis* [38]. Soluble mannan inhibited phagocytosis of yeast and an IL-17 response to yeast in vitro. MR^−/−^ mice did not make as much IL-17 or IFN-γ in response to immunization as wild-type mice and were not protected as well by immunization, indicating that the mannose receptor was important for effective innate immunity shaping of acquired immunity. However, the relative importance of the mannose receptor compared to Dectin-2 and Dectin-3 is not clear.

#### 4.1.3. TLR

There are no published studies about the potential role of TLRs in immunity to blastomycosis.

#### 4.1.4. Human Innate Immunity

With the recent immigration of Hmong refugees into the endemic area, it was realized that they had a greater risk of symptomatic blastomycosis than did European-Americans living in the same county [39]. Recently a genome-wide analysis of seven Hmong patients with clinically significant blastomycosis identified 25 polymorphisms in proximity to the IL-6 gene or AS-IL6, a long non-coding RNA that was recently reported to be involved in IL-6 production. These variants were associated with lower levels of IL-6 production and with fewer antigen-specific T_H_17 cells [40]. Interestingly, IL-6^−/−^ mice were not protected by a live vaccine from subsequent challenge with virulent *B. dermatitidis*, but unvaccinated IL-6-deficient mice were not more susceptible to primary infection. There is no evidence in human beings that polymorphisms in innate immune receptors influence innate resistance to blastomycosis.

### 4.2. Coccidioides spp.

*Coccidioides* spp. grow in a mycelial form in the environment and form arthroconidia as the reproductive phase. These can spread in the environment to form more mycelia or differentiate into a tissue phase known as spherules, when infecting animals. Almost all infections occur via the respiratory route. In immunocompetent people, infection causes respiratory symptoms, varying from mild, self-limited illness to severe pneumonia. Significant symptoms can occur 30–50% of the time [41]. Some studies in the hyperendemic region have reported that 40% of the population has been infected [41] and coccidioidomycosis is a relatively common cause of community-acquired pneumonia in the endemic area [42]. Dissemination beyond the lungs can occur in immunocompetent people, but it occurs more frequently in the immunocompromised or in special circumstances such as the third trimester of pregnancy.

#### 4.2.1. CLR

The role of Dectin-1 was first explored using a cell line engineered to express high levels of Dectin-1 [43]. Cells expressing high levels of Dectin-1 made more pro-inflammatory cytokines in response to spherules than control cells. β-glucan extracted from spherules stimulated cells to make pro-inflammatory cytokines in a Dectin-1-dependent manner as well. Furthermore, an anti-Dectin-1 monoclonal antibody substantially reduced the pro-inflammatory cytokine response of elicited macrophages to spherules. These data suggest that Dectin-1 is an important receptor for the pro-inflammatory cytokine response to spherules.

Other studies also indicated the importance of Dectin-1 in the mouse innate immune response to spherules. Elicited peritoneal macrophages from Dectin-1^−/−^ mice made much lower amounts of the pro-inflammatory cytokines TNF-α, IL-6, and MIP-2 in response to spherules than peritoneal macrophages from control animals [44]. However, when the response of bone marrow-derived dendritic cells (BMDCs) was measured, the results were more nuanced. The IL-6, IL-10, and granulocyte-macrophage colony-stimulating factor (GM-CSF) responses to spherules were largely, but not entirely, dependent on Dectin-1, but IL-1 and TNF-α production was independent of Dectin-1.

The absence of Dectin-1 also had a dramatic effect on the course of experimental infection. Bronchial alveolar lung fluid (BALF) from Dectin-1^−/−^ mice obtained after intranasal infection contained significantly lower concentrations of protective T_H_17 and T_H_1 cytokines than BALF from infected control mice. T_H_17 cytokines are required for successful immunization to *Coccidioides* spp., so the involvement of Dectin-1 in the production of these cytokines may be part of a protective immune response.

Dectin-1^−/−^ mice were also more susceptible to *Coccidioides immitis* infection as measured by quantitative culture. Dectin-1^−/−^ C57BL/6 mice had slightly higher numbers of spherules in their lungs compared with controls and substantially higher numbers in the spleens, suggesting that the dissemination of infection was influenced by Dectin-1 [44]. Murine susceptibility to experimental coccidioidomycosis is greatly influenced by the genetic background of the animal: C57BL/6 mice (the strain with the Dectin-1^−/−^ deletion) are relatively susceptible to infection and DBA/2 mice are relatively resistant [45]. Therefore, the influence of the Dectin-1^−/−^ genotype may not be fully appreciated in C57BL/6 mice because even without targeted mutations they are already relatively susceptible to infection, even when Dectin-1 is present. To address this issue, Dectin-1^−/−^ C57BL/6 mice were crossed with resistant DBA/2 mice, and offspring from the F2 generation that were homozygous for Dectin-1^−/−^ were identified by PCR and compared to littermates with an intact Dectin-1. In this comparison, the deleterious effect of the Dectin-1 knockout (KO) mutation was more obvious in both the lung and spleen.

Whether differences in Dectin-1-mediated responses play a role in determining the relatively high innate resistance of DBA/2 mice compared to the relatively poor resistance of the C57BL/6 mouse strain has also been addressed [46]. Peritoneal macrophages from DBA/2 mice made more pro-inflammatory cytokines than those from C57BL/6 mice and the response was dependent on Dectin-1 and TLR-2. Although the level of Dectin-1, as determined by RT-PCR, was identical in the two strains of mice, the Dectin-1 product was about 100 bp shorter in the C57BL/6 mice than in the DBA/2 mice, which was the result of alternative splicing of *Clec7a* by C57BL/6 mice. This deletion excluded exon 3, which codes most of the extracellular stalk. A cell line transfected with the C57BL/6 isoform of truncated Dectin-1 made half as much TNF-α as those transfected with full length Dectin-1 when stimulated with killed spherules. Furthermore, in BXD recombinant inbred mouse strains, there was a strong correlation between expression of full-length Dectin-1 and resistance to infection, which is further evidence supporting the importance of full-length Dectin-1 for resistance to infection. It is intriguing that mouse Dectin-1 is found on chromosome 6, very close to a region that has previously been identified as an important gene for resistance to infection in mouse [47], which may be additional evidence of the importance of Dectin-1 in genetically determined resistance.

The role of murine Dectin-2 and the mannose receptor on cytokine secretion in vitro and resistance to infection in vivo was studied using Dectin-2 and MR^−/−^ mice. Both peritoneal macrophages and BMDCs from MR^−/−^ mice made lower levels of pro-inflammatory cytokines in vitro in response to spherules compared to controls. However, MR^−/−^ mice were no more susceptible to experimental intranasal *C. immitis* infection than control mice [48]. It is not clear why there is a disparity between the importance of the MR for cell activation in vitro and the observation that the MR does not play a role in resistance to infection.

The findings with Dectin-2 KO mice were more complex. Peritoneal macrophages from Dectin-2^−/−^ mice made extremely poor pro-inflammatory cytokine responses to spherules in vitro compared to controls. However, BMDCs from Dectin-2^−/−^ mice made identical protective cytokine responses in vitro to spherules, but lower amounts of IL-10. Dectin-2^−/−^ mice were no more susceptible to experimental coccidioidomycosis than controls [48]. To address the question of whether these two C-type receptors were redundant, mice lacking both MR and Dectin-2 were also tested for resistance to infection. Mice with neither of these two receptors were no more susceptible to infection than controls, indicating that these two receptors were not redundant and do not play a critical role in host defense to coccidioidomycosis in mice, despite the role they play in vitro [48].

Dectin-2 seems to play a different role in the acquired immune response to *Coccidioides* spp. [35]. Immunization of Dectin-2^−/−^ mice with a live, attenuated mutant led to lower levels of IL-17 as well as IFN-γ. Immunized Dectin-2^−/−^ mice were more susceptible to infection than immunized controls. In contrast, elimination of the Mincle receptor had no effect on acquired immunity.

#### 4.2.2. TLR

Elicited peritoneal macrophages from TLR-2^−/−^ mice made a very poor pro-inflammatory cytokine response to spherules compared to macrophages from control mice [43]. In contrast, macrophages from mice with a point mutation in TLR-4 producing a non-functional TLR-4 made an identical response to spherules, compared to control mice, suggesting that TLR-4 is not an important receptor for spherules. This observation is consistent with a previous study of genetically determined resistance to *C. immitis* in mice, in which a TLR-4 null mutation was found to have no effect on resistance to infection [45].

MyD88 is a signal transducing molecule that is downstream of the TLR receptors, TLR-2, TLR-4, TLR-7, TLR-9 as well as IL-1R-1 and IL-18R [49]. TIR-domain-containing adapter-inducing interferon-β (TRIF) s another signal transducing molecule that is downstream of TLR-3 and TLR-4 [50]. Since TLR-2 is important for the cytokine response of elicited peritoneal macrophages to spherules, it was predicted that peritoneal macrophages from MyD88/TRIF^−/−^ mice would make poor cytokine responses to spherules, which was the case. However, BMDCs from both MyD88^−/−^ and MyD88/TRIF^−/−^ mice made as much TNF-α, IL-6, and IL-10 in response to spherules as controls [51].

Despite the unchanged cytokine response by BMDCs, MyD88^−/−^ and MyD88/TRIF^−/−^ mice were more susceptible than controls to coccidioidomycosis. However, neither TLR-2^−/−^ nor TLR-4^−/−^ mice were more susceptible to infection, in contrast to the poor cytokine responses of peritoneal macrophages to spherules from TLR-2^−/−^ mice. Since MyD88 is also a signal transducing molecule for the IL-18 receptor and the IL-1 receptor, the effects of these receptors on resistance to infection were studied. IL-18R^−/−^ mice were no more susceptible to infection than control mice, but IL-1 receptor^−/−^ mice were much more likely to develop disseminated disease. These experiments suggest that signaling by IL-1 may play a critical role in innate immunity to coccidioidomycosis in mice.

#### 4.2.3. Human Innate Immunity

There is in vitro evidence that the mannose receptor plays a role in recognition of *Coccidioides posadasii* spherules by human dendritic cells (DCs), since spherule binding and cytokine production can be inhibited by mannan [52,53].

A small number of people with disseminated coccidioidomycosis have been found to have mutations in the IFN-γ receptor [54] or the IL-12 receptor [55], evidence for the importance of IFN-γ and CD4 T_H_1 cells in immunity to coccidioidomycosis. A gain-of-function mutation in the signal transducer and activator of transcription 1 (STAT1). IFN-γ/IL-12 signaling protein that dysregulates signaling has also been observed in two patients with disseminated disease [56]. All these mutations have a profound effect on T_H_1 and T_H_17 immune responses, which are critical host responses. People who have low numbers of T cells or poor T-cell function because of infections, such as HIV/AIDS, or immunosuppressive medical therapies, are also at a high risk for severe infection and/or disseminated infection [57,58].

In addition to these predispositions, there is a marked effect of ethnicity on the course of infection. African Americans are significantly more likely to develop disseminated disease than those of European descent [59,60]. This observation indicates that there are some genetic factors involved in resistance to infection that are currently unknown. Since polymorphisms in innate immune receptors play a role in the susceptibility of inbred mice to coccidioidomycosis, investigating these polymorphisms in human beings is an attractive idea.

### 4.3. Cryptococcus spp.

*Cryptococcus neoformans* is found throughout the world, usually associated with pigeon excreta [61]. *Cryptococcus gattii* is found primarily in warmer climates, most frequently associated with eucalyptus trees. The number of asymptomatic or self-limited cryptococcal infection is unclear, but recent data suggests that they are common [62]. The organism is inhaled, but symptomatic pneumonia is uncommon, while most symptomatic patients present with meningitis. Both *C. neoformans* and *C. gattii* infect people, but about 95% of human infections are caused by *C. neoformans* [61] *C. neoformans* infections cause disease primarily in people with CD4 T-cell deficiencies such as AIDS and lymphomas. *C. gattii* appears to be better able to cause disease in non-immunocompromised people, but it too can infect those with T-cell deficiencies [63]. Unfortunately, the majority of studies of innate resistance in mice have been done with *C. neoformans*, and it is not clear whether the results reflect innate immunity to *C. gattii* infections too. Overall, most cases of cryptococcal meningitis occur in immunocompromised hosts [64]. Resistance to *C. neoformans* clearly requires CD4^+^ T-cells and AIDS is the most common predisposing illness, but the innate immune system also plays an important role. Increasingly, it is being recognized that the development of autoimmune neutralizing antibodies to protective cytokines such as GM-CSF and IFN-γ predisposes to cryptococcal pneumonia [65,66].

#### 4.3.1. Capsule Receptors

The large, complex carbohydrate capsule is the most important virulence factor by virtue of preventing phagocytosis and intracellular killing. Acapsular mutants of both *C. neoformans* and *C. gattii* are avirulent [7,67]. GXM is the major component of the capsule and it is an immunosuppressant, inhibiting T_H_1 responses and dendritic cell maturation in human blood cells and mice [68,69]. There is compelling evidence that the mannose receptor and the Fc receptor II (FcRγ) are functional receptors for GXM [70]. FcRγ^−/−^ mice are more susceptible to infection than controls in an intravenous infection model and have more organisms in their organs, indicating that this receptor is critical for innate resistance in mice, although this study is somewhat flawed by systemic lupus erythematosus that the FcRγ deletion causes [71].

#### 4.3.2. CLR

CARD9 deficiency in mice makes them more susceptible to cryptococcal infection and leads to impaired production of IFN-γ by T cells in the lungs, suggesting that a CLR is required to generate a proper innate immune response [72]. However, neither Dectin-1 nor Dectin-2 play an important role in macrophage binding of spores and play a modest role in the phagocytosis of spores at most [73].

Furthermore Dectin-1- or Dectin-2-deficient mice are not more susceptible to infection than controls, although Dectin-2^−/−^ mice make a larger T_H_2 immune response [73,74,75]. It was recently reported that Dectin-3 recognizes the principle component of the capsule GXM of three of the four serotypes of *Cryptococcus* spp. (*C. neoformans* A and AD; *C. gatti* B, but not C) and Dectin-3-deficient mice are more susceptible to those three serotypes [76]. The need for Dectin-3 may explain why CARD9-deficient mice are more susceptible to infection. The mannose receptor is involved in macrophage binding of *C. neoformans*, but its role in determining resistance is not known. The potential role of other CLRs in innate resistance to infection, such as Mincle, DC-SIGN, or galectin-3, has not been investigated. However, Mincle does not appear to be required for phagocyte binding of spores [73].

#### 4.3.3. TLR

The role of TLR-2 and TLR-4 is controversial, but there is agreement that MyD88 is important. Macrophages from mice with a TLR-2 or MyD88 deletion make less TNF-α in vitro in response to live *C. neoformans* yeast than controls [77]. In one study, MyD88 and TLR-2 appeared to play a role in resistance to pulmonary cryptococcal infection in mice, while TLR-4 did not [77]. The effect on innate resistance was seen in both survival and quantitative cultures, especially cultures of the spleen and brain. Another group also found that both TLR-2 and MyD88 influenced survival, but their interpretation of their data was that the influence of TLR-2 was relatively minor, while MyD88 played a larger role. [78]. A third study of pulmonary cryptococcal infection found that TLR-2 was not important for resistance to infection, using colony counts and immunologic responses as criteria [79]. It is possible that differences in the *Cryptococcus neoformans* strains used to challenge the mice might play a role in this discrepancy.

Other receptors dependent on MyD88 signaling have also been studied. TLR-9, IL-18R, and MyD88 were found to play an important role in resistance to pulmonary challenge, while IL-1R was not [80]. The importance of IL-18R is consistent with the importance of IL-18 in resistance to infection [81]. One conclusion that can be drawn from these studies is that one or more MyD88-dependent receptors are important for innate resistance to *C. neoformans* in mice.

#### 4.3.4. Human Innate Immunity

The most common predisposition to cryptococcal infection is HIV/AIDS, and effective treatment of HIV has reduced the incidence of this infection dramatically. However, there are other predispositions to infection. In human studies, polymorphisms in FcγR and mannose binding lectin (MBL) have been associated with increased susceptibility to infection in both HIV non-infected [82] and HIV infected patients [83]. One FcRγ variant associated with increased susceptibility bound Ig-*C. neoformans* immune complexes more efficiently than the wildtype, which the authors suggest might lead to more heavily infected phagocytic cells [82]. A number of different FcRγ polymorphisms have been associated with increased susceptibility to disease [84]. These human observations are consistent with the importance of the FcRγ in the response to GXM and further support the pivotal role of this receptor for innate immunity to this organism.

### 4.4. Histoplasma spp.

*Histoplasma* spp. grow as a mold in moist soil, especially soil contaminated with bird or bat droppings [85]. The organism is highly endemic in the soil of Mississippi and Ohio River valleys in the United States, and endemic in a number of regions in Central America and South America, but it also occurs in many scattered areas across the world. After inhalation, the spore differentiates into the yeast form. Infection is usually asymptomatic but some people develop pneumonia, which is typically self-limited, but can be very severe. Others develop a serious chronic disease, such as a disseminated infection or fibrotic lung disease. People who are unable to make a normal T-cell immune are more likely to develop disseminated disease than those with an intact immune system [86].

#### CLR

(a) Receptors for Phagocytosis:

*Histoplasma* spp. yeasts are readily phagocytosed and usually contained within the phagosome when the macrophages are activated [87]. Activated macrophages can inhibit yeast growth and the organism can be killed after phagosomal/lysosomal fusion. Early studies reported that the CD18 family of integrin receptors, complement receptor 3 (CR3), lymphocyte function associated antigen-1 and p150/95, were important for yeast adherence to human monocytes [88]. A recent study of phagocytosis in mouse peritoneal macrophages confirmed the importance of CR3 and Dectin-1, but ruled out a role for TLR-2, TLR-4, MR, DC-SIGN/SIGNR1, FcRγ and Dectin-2 in experiments using monoclonal blocking antibodies [89].

(b) CLR Involved in Cytokine Production and Innate Resistance to Infection:

Dectin-1 also collaborated with CR3 in triggering the cytokine response to *Histoplasma capsulatum* yeasts, and Syk tyrosine kinase was involved in phagocytosis and cytokine production [90]. Macrophage production of cytokines in response to *H. capsulatum* yeast could be blocked by cytochalasin, suggesting that phagocytosis of the organism was required for this response. The cytokine response could also be blocked by pretreatment of the yeast with sialidase, suggesting that macrophage binding to sialic acid might be involved, possibly mediated by a sialic acid-binding immunoglobulin-type lectin. Antibodies to CR3, Dectin-1, and TLR-2 all inhibited macrophage cytokine responses to the yeast.

Dectin-1 can be a major receptor for stimulation of cytokine production by macrophage cell lines stimulated with *H. capsulatum* yeast that lacks the enzyme, α-(1,3)-glucan synthase 1 (AGS1) [91]. However, chemotype II strains of *H. capsulatum* have AGS1, which produces a layer of α-(1,3)-glucan above the β-(1,3)-glucan layer and masks Dectin-1 recognition of the yeast and stimulation of macrophage to make cytokines [91]. It would seem likely that chemotype I strains that lacked AGS1 would be less virulent than chemotype II, but chemotype I and chemotype II strains are equally virulent. This apparent discrepancy has been resolved by the discovery that chemotype I strains produce a beta-glucanase (ENG-1), an extracellular enzyme that hydrolyzes β-(1,3)-glucan linkages [92]. A reduction in the transcription of this enzyme in yeasts by inhibitory RNA (RNAi) resulted in yeasts that were bound by soluble Dectin-1 better than controls, as would be expected since the amount of exposed β-(1,3)-glucan on the cell surface was increased. Furthermore, RNAi-transfected yeasts expressing little ENG-1 stimulated mouse macrophages and dendritic cells to make more pro-inflammatory cytokines. *H. capsulatum* producing little ENG-1 were much less pathogenic than controls in a pulmonary challenge; 100–1000 fewer RNAi-producing organisms were recovered compared to controls and all infected mice survived. This difference in pathogenicity was seen in Dectin-1^+/+^ mice, but not in Dectin-1^−/−^ mice, indicating that Dectin-1 interaction with β-(1,3)-glucan was responsible for the effect. This is the strongest evidence that Dectin-1 could play a critical role in the innate immune response to *H. capsulatum* yeast if β-glucan is expressed on the cell surface, but is of little importance for innate immunity to wild-type *H. capsulatum*.

The resistance to experimental histoplasmosis in mice is greatly influenced by the mouse genetic background. The genes which are the most important for genetically determined resistance are in the major histocompatibility complex (H-2) [93]. However, at least one modifying gene outside the H-2 complex was predicted. Other studies of genetically determined resistance have suggested that a modifying gene maps to chromosome 6, consistent with a possible role of genes within the C-type receptor [94]. However, neither of these studies identified Dectin-1 as a critical determinant of genetically determined resistance.

Dectin-2, in concert with Dectin-1, is an important receptor for *H. capsulatum* yeast for initiation of NLRP3 inflammasome activation in DCs [95]. The inflammasome is important for processing pro-IL-1 cytokines into bioactive molecules. NLRP^−/−^ mice are more susceptible to infection than controls, indicating that this pathway is important for natural resistance. In addition, BMDCs from Dectin-2^−/−^ mice made less IL-1β than controls in vitro. These authors did not test the effect of Dectin-2-targeted deletion on the course of primary infection. However, Dectin-2 plays a very similar role in acquired immunity to *H. capsulatum* as it does in *B. dermatitidis* and *Coccidioides* spp. and is important for immunization [35].

(c) TLR:

Mice with targeted deletions of MyD88 are more susceptible to *H. capsulatum* than controls as measured by survival and colony counts in lung and spleen [96]. In contrast, Dectin-1^−/−^ mice and IL-1R^−/−^ mice were no more susceptible to infection than controls, despite the fact that the infecting organism was a chemotype I strain. The amount of pro-inflammatory cytokines, including IFN-γ and IL-17, in the lungs of infected mice and the number of T cells in the lungs were lower in the MyD88^−/−^ animals than controls. These results suggest that a receptor upstream of MyD88 is important for the innate immune response.

TLR7 and TLR9 are important receptors for generating type-I interferon (IFN-I), IFN-γ, and IL-12 responses of dendritic cells to *H. capsulatum* yeasts [97]. Dendritic cells exposed to living (but not killed) *H. capsulatum* yeast produced a number of IFN-I-related genes. TLR-7/9^−/−^ (the double knockout mutant) or MyD88 mice made very little IFN-I in response to the yeast and mice lacking the IFN-γ receptor or TLR-7/9 were more susceptible to infection than controls as measured by survival and quantitative culture. This difference was most dramatic in the brain, an organ that is not usually cultured in experimental mouse infections, but can be infected in human disseminated histoplasmosis. Dendritic cells from TLR-7/9 mice did not produce IFN-γ nearly as well as dendritic cells from control mice in response to primed T-cells and the yeast. The effect of MyD88 deletion seemed more dramatic than the effect of TLR-7/9, but they were not compared directly.

(d) Humans:

Many of the same human mutations affecting cell-mediated immunity that increase susceptibility to coccidioidomycosis are also risks for disseminated histoplasmosis. Other genetic polymorphisms associated with resistance in humans have not been described.

### 4.5. Paracoccidioides spp.

Paracoccidioidomycosis is the most common endemic fungal infection in South America with most of the cases occurring in the Amazon basin. It is caused by a number of closely related species of *Paracoccidioides* spp. that grow as molds in the soil and as yeasts inside an animal host. Infection occurs by the respiratory route and illness is mostly limited to pulmonary involvement. Dissemination to the skin, mucous membranes, and a variety of other organs sometimes occurs, and it is more common in men than women and in people with an immunocompromised cell-mediated immune system [98].

#### 4.5.1. CLR

The importance of Dectin-1 was explored using Dectin-1^−/−^ mice. Elicited peritoneal macrophages from Dectin-1^−/−^ mice bound or ingested fewer yeasts than that of control animals and produced smaller amounts of pro-inflammatory cytokines and larger amounts of anti-inflammatory cytokines [99]. Dectin-1^−/−^ mice were also more susceptible to pulmonary infection as measured by quantitative culture of the lung and spleen as well as survival and had lower numbers of activated T-cells in their lungs. Finally, Dectin-1^−/−^ mice had lower numbers of pulmonary T_H_17 cells and higher numbers of regulatory T cells (Treg) cells in infected tissues. All these observations argue for an important role of Dectin-1 in innate and acquired resistance to paracoccidioidomycosis. In vitro studies in murine cells with polysaccharide Dectin-1 agonists and antagonists also suggested that Dectin-1 was an important innate immune receptor for the yeast [100]. A part of the mouse BMDC response to *Paracoccidioides brasiliensis* yeast is increased transcription of Dectin-1, which is also consistent with the importance of this receptor [101]. There have been no published studies of paracoccidioidomycosis in Dectin-2^−/−^ mice.

#### 4.5.2. TLR

In TLR-2^−/−^ mouse studies, the role of TLR-2 in paracoccidioidomycosis was less clear. In one study, the number of organisms in the lungs was slightly, but significantly, lower in TLR-2^−/−^ mice than in controls, but the survival curves were not significantly different. The authors believed that this discrepancy in results was due to increased inflammation in the TLR-2^−/−^ animals (they had more PMNs in their lungs), higher numbers of T_H_17 T-cells, and fewer Tregs compared with that of controls [102]. However, another interpretation of the data is that TLR-2 has a minimal effect on the course of the disease. In vitro studies have found that TLR-4 cooperates with Dectin-1 and mannose receptor to expand T_H_17 cells induced by *P. brasiliensis*-stimulated dendritic cells [103]. There are no published studies of experimental paracoccidioidomycosis in TLR-4^−/−^ mice.

The role of MyD88 in innate immunity to infection is controversial. In a study of pulmonary paracoccidioidomycosis, MyD88^−/−^ mice were shown to be more susceptible to disease as measured by survival and quantitative culture [104]. The differences in organism load were most significant in the spleen and liver. In infected mice, there were fewer CD4 T-cells in MyD88^−/−^ lungs and lower amounts of IL-17, a cell type and cytokine that are important for resistance. In contrast, another study found that MyD88^−/−^ mice were not more susceptible to paracoccidioidomycosis, as measured by quantitative culture, in an intravenous infection model [105]. Similar levels of cytokines and a similar frequency of IFN-γ-producing T cells were also found in MyD88^−/−^ and control mice. However, given that the pulmonary route of infection is more physiologic, it is more likely that MyD88 plays a role in resistance.

TLR-9 plays an important role in paracoccidioidomycosis in mice [106]. *P. brasiliensis* DNA stimulates mouse macrophages to make pro-inflammatory cytokines via the TLR-9 receptor. TLR-9^−/−^ mice were significantly more susceptible to intravenous infection than controls as measured by survival rates. The difference was seen within the first few days of infection. The TLR-9^−/−^ mice made a more vigorous inflammatory histologic and cytokine response to infection compared to controls. The organism loads were not reported. It should be noted again that the physiologic route of infection is pulmonary.

Genetically determined resistance to intraperitoneal infection has been described in mice [107]. The authors believed this to be a single, dominant, autosomal gene in the A/J strain (resistant) and B10.A/N strain (susceptible), but recombinant inbred strains derived from those two strains yielded intermediate resistance phenotypes, suggesting that more than one gene influences resistance. Other mouse strains also had intermediate resistance phenotypes. The resistance gene was not linked to the H-2 locus, but it has not been localized on the genetic map, so its proximity to any innate immune receptor is not known.

#### 4.5.3. Galectin

Galectins are a family of soluble β-galactoside-binding proteins that are widely expressed at sites of inflammation, infection, and tumor growth, and play a role in inflammation and immunity [108]. Galactin-3^−/−^ mice were used to study the roles of this soluble receptor in resistance to *P. brasiliensis* infection [109]. Galactin-3^−/−^ mice were more susceptible to infection than controls, as measured by survival and colony counts. The mice lacking galectin-3 made more T_H_2 cytokines than controls and induced the transcription of TLR-2, a receptor that is associated with IL-10 production, more efficiently. The mechanism for this observation is unclear, but galectin-3 is upregulated by *P. brasiliensis* infection in mice and directly inhibits the growth and budding of *P. brasiliensis*, as well as increasing the uptake of organisms by macrophages.

#### 4.5.4. Humans

In contrast to studies in mice, in vitro studies of human plasmacytoid dendritic cells using antibodies to MCL showed that Dectin-2 and Dectin-3 were important [110]. Dendritic cells ingested *P. brasiliensis* and inhibited the organism’s growth and this growth inhibition was blocked by anti-Dectin-2 and anti-Dectin-3. Anti-Dectin-1 and anti-Minicle antibodies did not block growth inhibition in this study. Dendritic cells also made TNF-α in response to *P. brasiliensis* yeast and that response was blocked by anti-Dectin-2 antibody or a pharmacologic inhibitor of Syk.

Human neutrophils produce both pro-inflammatory and anti-inflammatory cytokines including TNF-α in response to *P. brasiliensis* yeast. Anti-MR and anti-Dectin-1 monoclonal antibodies(mAb) both inhibited these responses. Anti-TLR-2 mAb was not tested alone, but the combination of anti-Dectin-1 and anti-TLR-2 was highly effective [111].

There have only been a small number of cases of disseminated paracoccidioidomycosis reported in patients with AIDS, but those patients have severe disease [112]. It is certainly possible that the relatively small number of patients is due to under-reporting. Polymorphisms in DC-SIGN and vitamin D receptor were associated with the development of oral lesions [113].

## 5. Evaluating the Data

Innate immune receptors and their signal transducing molecules clearly play an important role in the overall immune response to primary pathogenic fungi. The evidence for this comes from a variety of different types of experiments, each with strengths and limitations. The best controlled studies are experimental infections in mice with targeted deletions in a variety of genes. To be most informative, the effective of the targeted deletion should be determined both in the innate phase of infection and the acquired immune response to immunization with a live, attenuated mutant. The effects of the deletion on cytokine responses and macrophage, DC, and lymphocyte responses might also give an insight into the mechanisms of immunity and immune defects, and reveal circumstances where responses were affected differently by targeted deletions. In vitro studies with cells from mice with targeted deletions are also useful, but knowing what types of responses are required for protective immunity is not always easy. Measuring the ability of phagocytic cells to ingest and inhibit the growth of the organism intuitively seems to reflect a protective response. Changes in the production of cytokines is more problematic to relate to resistance to infection. In a number of instances, receptors involved in cytokine production do not have the predicted effects on resistance to infection. Some receptors play a role in producing one cytokine more than others and both pro-inflammatory and anti-inflammatory cytokines could play a protective role under certain circumstances. Furthermore, a limited number of cytokines is usually measured, so the overall effect of a targeted deletion is not clear. In addition to studies in mice with targeted deletions, studies in mouse strains with differences in innate susceptibility can be very informative. If differences in susceptibility correlate with polymorphisms in the innate immune response, and especially if the polymorphism maps to the same region of the chromosome as susceptibility, this is strong evidence that the polymorphism is important for innate immunity.

In our opinion, studies of the cellular response to an organism in vitro using anti-receptor antibodies or soluble carbohydrate receptor antagonists are less informative. The choices of what cells to study and the responses to be measured have the same issues as mentioned above. Furthermore, the amount of an antibody needed and the ability of the antibody to block receptor–ligand interaction are potential issues. In addition, in some cases, antibody-mediated clustering of receptors may have unexpected consequences. Soluble inhibitors are even more problematic. The concentration needed to block activation is variable and it is often not clear whether the inhibitor is specific for the receptor being studied. In addition, some soluble carbohydrate preparations are not pure and may have off-target effects for this reason.

Studies in human beings are the most informative for medical research. Experimental studies are limited to in vitro experiments, with the limitations mentioned above. Observations of genetically determined resistance can be very useful for inferring important host resistance mechanisms. A major limitation of human genetic studies for endemic fungi is that the fraction of the population that is exposed to the infectious agent is relatively low, which makes recognizing increased susceptibility to infection more difficult. The importance of CARD9 for resistance to *Candida* spp. infections might not have been seen if only a few people were at risk for infection by these organisms. Nevertheless, polymorphisms in human genes can be identified as associated with susceptibility to infection when they are found in patients with unexpectedly severe forms of infection, especially when they are familial and inherited in a Mendelian manner. If susceptibility is a polygenic trait, then variants with small effects will be difficult to identify as those studies require large numbers of cases and known infected controls with self-limited infections.

## 6. Summary

Despite all these caveats, we can draw some tentative conclusions from the experiments discussed here. Table 2 shows some conclusions from mouse studies. The overall conclusion is that more than one type of receptor plays an important role in resistance to these primary pathogenic fungi. More studies of the innate immune receptors to fungal infections in mice, especially systematic studies comparing the importance of one receptor to another in a single model of the innate immune receptors to fungal infections in mice, and human in vitro studies and genetic studies will clearly improve our understanding of this important topic.

## Figures and Tables

**Table 1:** Fungal pattern recognition receptors and their ligands.

	Pattern Recognition Receptor	Ligand	Reference
CLR ^a^	Dectin-1	β-(1,3)-glucan	[8]
	Dectin-2	High mannose	[9]
	Dectin-2/Dectin-3	α-mannan	[10]
	Mannose receptor	N-linked mannan	[11]
	Mannose receptor	Chitin	[12]
	Mincle ^b^	α-mannan	[13]
	Galectin-3	β-(1,2)-mannoside	[14]
	DC-SIGN	N-linked mannan	[15]
Others	NOD-2 ^c^	Chitin	[12]
TLR ^d^	TLR-2	Phospholipomannan	[16]
	TLR-2	β-(1,6)-glucan	[11]
	TLR-4	O-linked mannans	[11]
	TLR-7	ssRNA^e^	[17]
	TLR-9	CpG DNA	[18]
	TLR-9	Chitin	[12]

Note: Some of the pattern recognition receptors and ligands that are involved in innate resistance to fungal infections. The biological responses elicited by receptor–ligand binding are discussed in the text. ^a^ C-type lectin receptors; ^b^ Macrophage inducible Ca^2+^-dependent lectin receptor; ^c^ Nucleotide-binding oligomerization domain-containing protein 2; ^d^ Toll-like receptors; ^e^ Single stranded RNA.

**Table 2:** Summary of innate immune receptors in fungal infections.

Organism	Innate Resistance	Acquired Response
*Blastomyces* spp.	CR3, CARD9-dependent	CARD9-dependent Dectin-2, Dectin-3, MR
*Coccidioides* spp.	Dectin-1 MyD88-dependent IL-1R	CARD9-dependent Dectin-2
*Cryptococcus* spp.	CARD9-dependent Dectin-3, FcRγ MyD88-dependent TLR-2, IL-18R	?
*Histoplasma* spp.	MyD88-dependent TLR-7/9 ^a,b^	CARD9-dependent Dectin-2
*Paracoccidioides* spp.	Resistance - Dectin-1 MyD88-probably TLR-2 Galectin	?

Conclusions about the receptors involved in innate resistance to infection and the immune response to infection. They are based on in vivo data reviewed in this paper. However, the effects of many receptors on innate resistance and acquired immunity have not been tested. ^a^ Receptors involved in phagocytosis are CR3 and Dectin-1; ^b^ receptors involved in cytokine production are Dectin-1, Dectin-2, TLR-2, and CR3. CR3; complement receptor 3; CARD9: **c**aspase recruitment domain family, member 9; MyD88: Myeloid differentiation primary response 88; FcRγ: Fcγ receptor; TLR-2: toll like receptor 2; IL-18R: IL-18 receptor.
